# Distinctive detection of insulinoma using [^18^F]FB(ePEG12)12-exendin-4 PET/CT

**DOI:** 10.1038/s41598-021-94595-6

**Published:** 2021-07-22

**Authors:** Takaaki Murakami, Hiroyuki Fujimoto, Keita Hamamatsu, Yuki Yamauchi, Yuzo Kodama, Naotaka Fujita, Junji Fujikura, Yoichi Shimizu, Yuji Nakamoto, Hiroyuki Kimura, Hideo Saji, Nobuya Inagaki

**Affiliations:** 1 Department of Diabetes, Endocrinology and Nutrition, Graduate School of Medicine, Kyoto University, 54 Kawahara-cho, Shogoin, Sakyo-ku, Kyoto, 606-8507 Japan; 2 Radioisotope Research Center, Agency of Health, Safety and Environment, Kyoto University, Kyoto, Japan; 3 Department of Gastroenterology, Hyogo Prefectural Amagasaki General Medical Center, Amagasaki, Japan; 4 Department of Gastroenterology, Graduate School of Medicine, Kobe University, Kobe, Japan; 5 Department of Diagnostic Imaging and Nuclear Medicine, Graduate School of Medicine, Kyoto University, Kyoto, Japan; 6 Department of Analytical and Bioinorganic Chemistry, Kyoto Pharmaceutical University, Kyoto, Japan; 7 Department of Patho-Functional Bioanalysis, Graduate School of Pharmaceutical Sciences, Kyoto University, Kyoto, Japan

**Keywords:** Endocrinology, Endocrine cancer, Neuroendocrine cancer

## Abstract

Specifying the exact localization of insulinoma remains challenging due to the lack of insulinoma-specific imaging methods. Recently, glucagon-like peptide-1 receptor (GLP-1R)-targeted imaging, especially positron emission tomography (PET), has emerged. Although various radiolabeled GLP-1R agonist exendin-4-based probes with chemical modifications for PET imaging have been investigated, an optimal candidate probe and its scanning protocol remain a necessity. Thus, we investigated the utility of a novel exendin-4-based probe conjugated with polyethylene glycol (PEG) for [^18^F]FB(ePEG12)12-exendin-4 PET imaging for insulinoma detection. We utilized [^18^F]FB(ePEG12)12-exendin-4 PET/CT to visualize mouse tumor models, which were generated using rat insulinoma cell xenografts. The probe demonstrated high uptake value on the tumor as 37.1 ± 0.4%ID/g, with rapid kidney clearance. Additionally, we used *Pdx1-Cre;Trp53*^*R172H*^*;Rb*^*f/f*^ mice, which developed endogenous insulinoma and glucagonoma, since they enabled differential imaging evaluation of our probe in functional pancreatic neuroendocrine neoplasms. In this model, our [^18^F]FB(ePEG12)12-exendin-4 PET/CT yielded favorable sensitivity and specificity for insulinoma detection. Sensitivity: 30-min post-injection 66.7%, 60-min post-injection 83.3%, combined 100% and specificity: 30-min post-injection 100%, 60-min post-injection 100%, combined 100%, which was corroborated by the results of in vitro time-based analysis of internalized probe accumulation. Accordingly, [^18^F]FB(ePEG12)12-exendin-4 is a promising PET imaging probe for visualizing insulinoma.

## Introduction

Insulinoma is a rare pancreatic neuroendocrine neoplasm (PanNEN) and causes hypoglycemia owing to insulin oversecretion^1^. It is the most common cause of endogenous hyperinsulinemic hypoglycemia in adult patients without diabetes and might play a part in the multiple endocrine neoplasia type 1 (MEN1) syndrome^2^. Because surgical resection is the only curative treatment for insulinoma, accurate preoperative tumor localization is essential for the determination of the precise area of resection in the pancreas^3,4^. However, determination of localization remains challenging because of the small sizes of these tumors^5,6^. Whereas the conventional imaging methods such as ultrasonography, computed tomography (CT), and magnetic resonance imaging have proven to display low sensitivity, selective arterial calcium stimulation test and endoscopic ultrasound are invasive and operator-dependent^6,7^. Additionally, somatostatin receptor (SSTR)-targeted imaging approaches have demonstrated improved sensitivities, but are highlighted by limitations because insulinomas, especially benign ones, lack reasonable expression levels of SSTR subtype 2 and 5^8,9^. Moreover, they do not reflect endocrine functions of the tumors, thereby making it impossible to differentiate insulinoma from other panNENs in cases with multiple panNENs.

Glucagon-like peptide-1 receptor (GLP-1R) is highly expressed on insulinomas^8,10^. Accordingly, GLP-1R-targeted imaging techniques such as single-photon emission computed tomography (SPECT) and positron emission tomography (PET) have recently emerged^11–13^. In particular, radiolabeled GLP-1R agonist exendin-4-based probes for PET imaging has been developed owing to the biological peptide stability of exendin-4 and the spatial resolution of PET. Although some exendin-4-based probes for SPECT and PET imaging showed their utilities in prospective clinical studies^13–17^, exendin-4-based probes with various chemical modifications are still being investigated for further improvement^13,18,19^. PEGylation is a well-established technique, which is known to improve pharmacokinetics and probe delivery to the targeted tumors^20,21^. However, exendin-4 probes conjugated with polyethylene glycol (PEG) have not been investigated for insulinoma detection.

Recently, we have reported that pancreas duodenum homeobox protein 1 (*Pdx1*) *Cre*-dependent pancreas-specific simultaneous deletion of retinoblastoma (*Rb*) gene with the induction of *p53* mutation induces multiple panNENs in islet cells^22^ and displays endogenous insulinoma and glucagonoma phenotype. Thus, we envisaged that the *Pdx1-Cre;Trp53*^*R172H*^*;Rb*^*f/f*^ mouse model could be promising in preclinical investigations of the sensitivity and selectivity of exendin-4-based probes for the specific detection of insulinoma and compared the imaging outcomes with conventionally constructed mice insulinoma model.

This study investigated the utility of a novel PEGylated exendin-4-based probe for PET imaging, [^18^F]FB(ePEG12)12-exendin-4, for insulinoma detection using *Pdx1-Cre;Trp53*^*R172H*^*;Rb*^*f/f*^ mice and conventional model mice bearing insulinoma xenografts.

## Results

### Biodistribution study of [^18^F]FB(ePEG12)12-exendin-4

In ex-vivo analyses using resected organs (n = 4 for each time point), the pancreatic uptake value of [^18^F]FB(ePEG12)12-exendin-4 was high at 30-min post-injection (11.3 ± 2.8%ID/g), remained high (12.7 ± 2.9%ID/g) at 60-min time point and was modest (7.9 ± 2.7%ID/g) at 150-min time point (Table 1). Kidney uptake of the probe was highest at 15-min posinjection and rapidly cleared by 150-min post-injection (2.2 ± 0.6%ID/g). Liver and spleen uptakes were constantly low, whereas lung showed relatively high uptake values.

**Table 1:** The biodistribution study of [^18^F]FB(ePEG12)12-exendin-4.

Post-injection time (min)	5	15	30	60	150	360
Blood	3.89 ± 0.23	1.45 ± 0.09	0.61 ± 0.05	0.25 ± 0.06	0.10 ± 0.04	0.01 ± 0.01
Pancreas	12.71 ± 3.60	11.15 ± 1.69	11.26 ± 2.82	12.66 ± 2.93	7.87 ± 2.74	7.87 ± 0.56
Kidney	77.14 ± 6.65	82.96 ± 5.87	40.01 ± 12.33	17.58 ± 4.38	2.24 ± 0.60	0.32 ± 0.09
Liver	1.12 ± 0.08	0.60 ± 0.01	0.43 ± 0.06	0.26 ± 0.03	0.10 ± 0.04	0.01 ± 0.01
Spleen	0.93 ± 0.08	0.61 ± 0.14	0.13 ± 0.10	0.16 ± 0.13	0.06 ± 0.05	0.03 ± 0.06
Lung	36.49 ± 5.71	33.50 ± 4.36	32.18 ± 9.52	30.98 ± 1.66	13.32 ± 2.70	3.22 ± 0.71

The data are calculated as uptake values of the organ per injected dose of the probe (%ID/g) and expressed as the mean ± SEM. The male ddY mice were used (n = 4 for each time point).

### Visualization of INS-1 xenografts in [^18^F]FB(ePEG12)12-exendin-4 PET/CT

**Figure 1:**
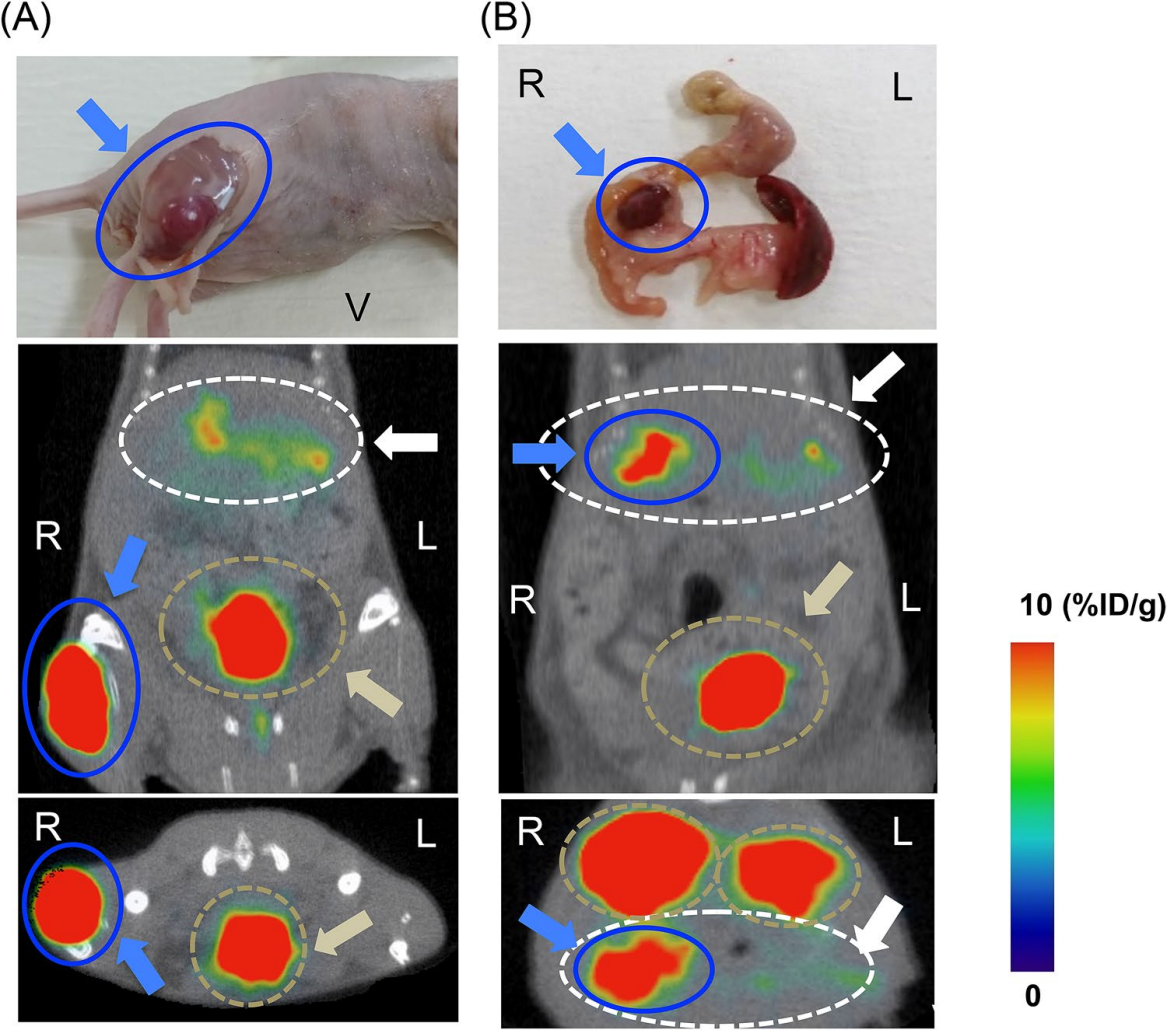
Representative [^18^F]FB(ePEG12)12-exendin-4 PET/CT images in INS-1 xenograft mice. INS-1 tumors of the right thigh (**A**) and pancreas head (**B**) were visualized in INS-1 xenograft BALB/c slc-*nu*/*nu* mice on [^18^F]FB(ePEG12)12-exendin-4 PET/CT images. In the upper panels, gross images of INS-1 tumors are shown (blue arrows and circles). Coronal and axial images are displayed in the middle and lower panels, respectively. Maximum to minimum intensity: red > orange > yellow > green > blue > black. The signals from the INS-1 tumors are blue arrows and circles. The signals from the pancreas are white arrows and dotted circles. The signals from the bladder are yellow–brown arrows and dotted circles. The signals from the kidney, yellow–brown arrows, and circles. %ID/g is the formula used in obtaining the uptake values of the region of interest per injected dose of the probe; L, left; R, right; V, ventral.

In the male BALB/c slc-*nu*/*nu* mice bearing INS-1 xenografts in the right thigh (n = 5), INS-1 tumors were properly visible on the [^18^F]FB(ePEG12)12-exendin-4 PET/CT images (Fig. 1A). The tumors reached a diameter of 11.0 ± 2.1 mm and a volume of 870.0 ± 31.1 mm^3^. Based on in-vivo imaging analysis, the tumor uptake values were 28.6 ± 4.4%ID/g, whereas the pancreatic uptake values were 12.7 ± 3.0%ID/g (n = 5, P < 0.01). The uptake ratios of the tumor per pancreas were 2.39 ± 0.39. In ex-vivo analysis, the tumor uptake values were 37.3 ± 4.5%ID/g and the uptake ratios of the tumor per kidney and blood were 3.30 ± 0.36 and 153.2 ± 12.5, respectively (n = 5). Moreover, in male BALB/c slc-*nu*/*nu* mice harboring INS-1 xenografts inside the pancreas (n = 3), the tumors grew to 7.6 ± 1.2 mm in diameter and 170.1 ± 40.3 mm^3^ in volume. [^18^F]FB(ePEG12)12-exendin-4 PET/CT yielded clear images of the INS-1 tumors (Fig. 1B) with high uptake value of 37.1 ± 0.4%ID/g (n = 3). The uptake ratios of the tumor per pancreas were 2.71 ± 0.27. In ex-vivo analysis, the tumor uptake values were 37.7 ± 4.4%ID/g and the uptake ratios of the tumor per kidney and blood were 3.44 ± 1.00 and 150.8 ± 15.0, respectively (n = 3).

### GLP-1R and SSTR2 mRNA expressions in pancreatic tumors of *Pdx1-Cre;Trp53*^*R172H*^*;Rb*^*f/f*^ mice

**Figure 2:**
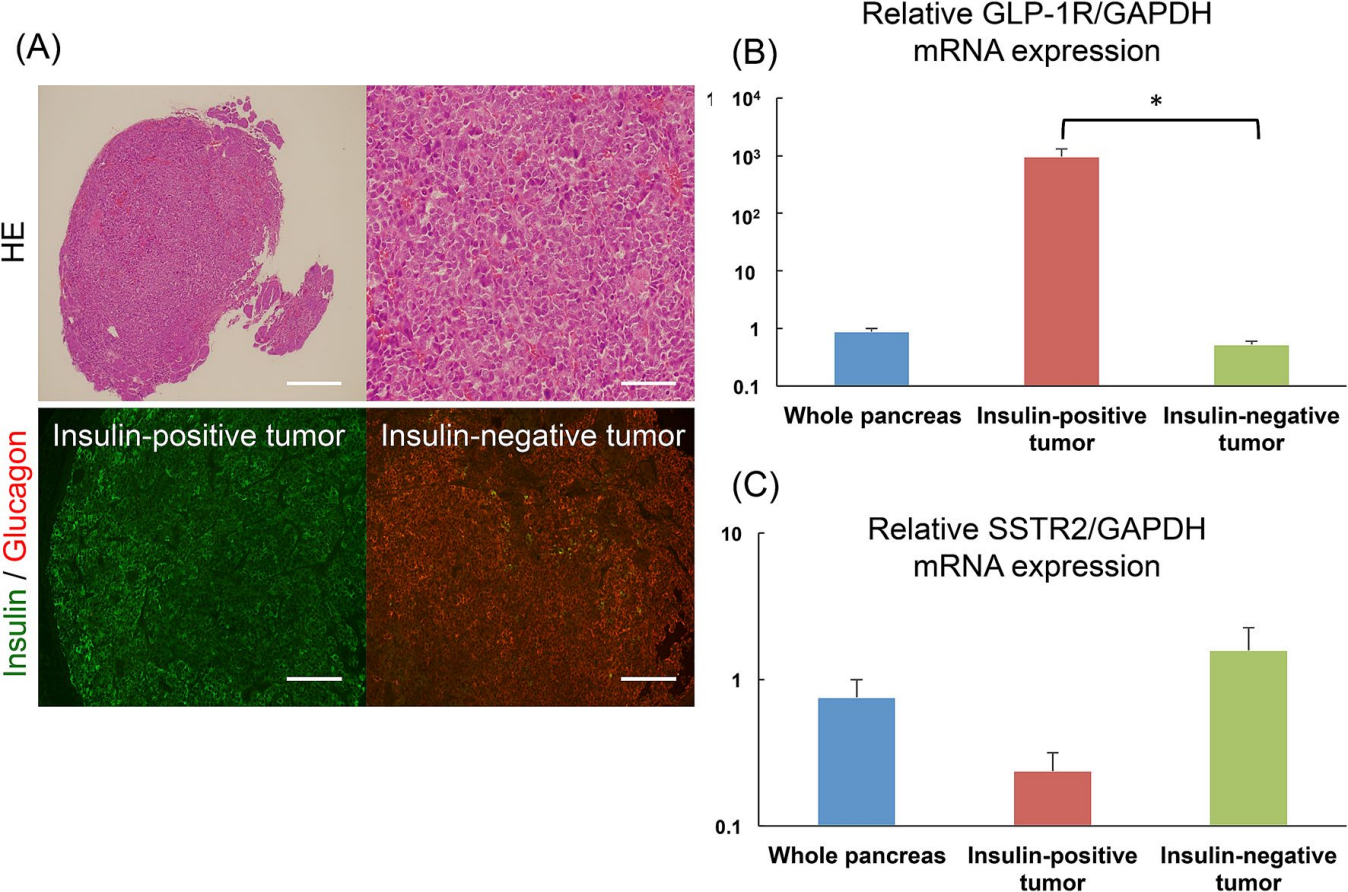
GLP-1R and SSTR2 mRNA expressions in pancreatic tumors of *Pdx1-Cre;Trp53*^*R172H*^*;Rb*^*f/f*^ mice. Representative microscopic images of resected pancreatic tumors of *Pdx1-Cre;Trp53*^*R172H*^*;Rb*^*f/f*^ mice are shown (**A**). Upper panels: Hematoxylin–Eosin (H&E), Scale bars indicate 500 μm in the left column and 200 μm in the right column, respectively; Lower panels: double immunofluorescent staining of insulin and glucagon. Insulin-positive tumor (left column) and insulin-negative tumor (right column). Scale bars indicate 200 μm. Relative GLP-1R (**B**) and SSTR subtype 2 (SSTR2) (**C**) mRNA expression levels of whole pancreas (n = 10), insulin-positive tumors (n = 6) and insulin-negative tumors (n = 6) are shown. The data are expressed as the mean ± SEM. *P < 0.05.

**Figure 3:**
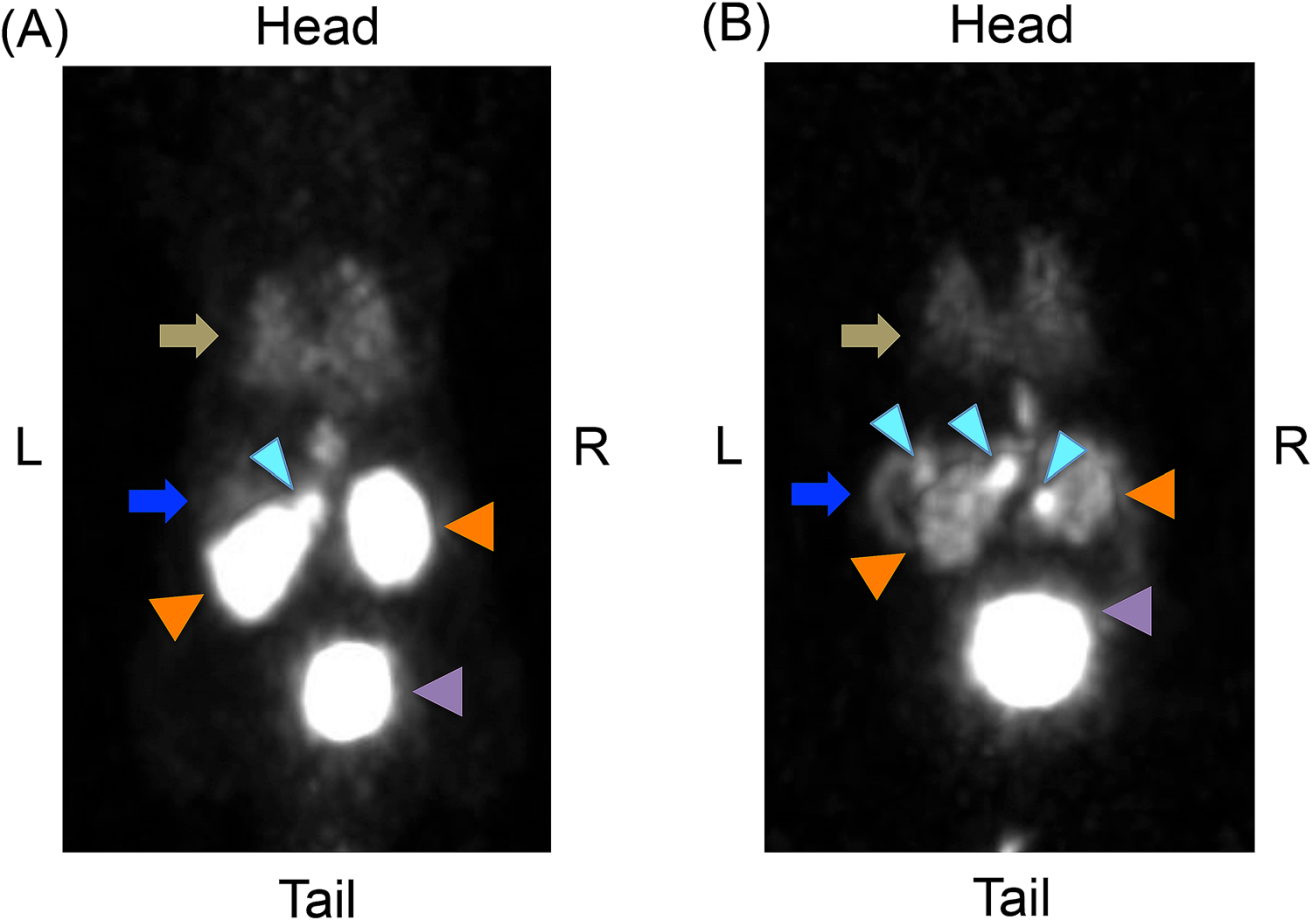
Representative maximum intensity projection PET images of [^18^F]FB(ePEG12)12-exendin-4. Maximum intensity projection (MIP) PET images of [^18^F]FB(ePEG12)12-exendin-4 in representative *Pdx1-Cre;Trp53*^*R172H*^*;Rb*^*f/f*^ mouse. MIP images were reconstructed at (**A**) 30-min post-injection and (**B**) 120-min post-injection of [^18^F]FB(ePEG12)12-exendin-4. MIP images indicated high tumor uptakes, sustained pancreas uptake and rapid kidney clearance of the probe. The signals from the tumors are represented by light blue arrowheads. Signals from the kidney, orange arrowheads; signals from bladder, mauve arrowheads; signals from the pancreas, blue arrows; signals from the lung, yellow–brown arrows. L, left; R, right.

For the resected pancreatic tumors of *Pdx1-Cre;Trp53*^*R172H*^*;Rb*^*f/f*^ mice with a non-fasting blood glucose level of < 80 mg/dL (n = 10), the relative GLP-1R and SSTR2 mRNA expression levels as well as anti-insulin and -glucagon antibody staining of each tumor are as follows: The immunohistochemical analysis of the insulin-positive tumors (n = 6) demonstrated glucagon-negative staining, whereas that of the insulin-negative tumors (n = 6) showed glucagon-positive staining (Fig. 2A), consistent with a previous report^22^. The expression levels of GLP-1R mRNA in insulin-positive tumors were significantly higher than those in insulin-negative tumors and the whole pancreas (Fig. 2B). In contrast, SSTR2 mRNA expression levels of insulin-positive tumors tended to be lower than those of insulin-negative tumors and whole pancreas (Fig. 2C), which was comparable with the clinical characteristics of insulinoma^8–10^. As for [^18^F]FB(ePEG12)12-exendin-4 PET imaging, maximum intensity projection images of a representative *Pdx1-Cre;Trp53*^*R172H*^*;Rb*^*f/f*^ mouse were shown in Fig. 3.

### [^18^F]FB(ePEG12)12-exendin-4 and [^68^Ga]DOTATOC PET/CT for pancreatic tumor detections in ***Pdx1-Cre;Trp53***^***R172H***^***;Rb***^***f/f***^ mice

**Figure 4:**
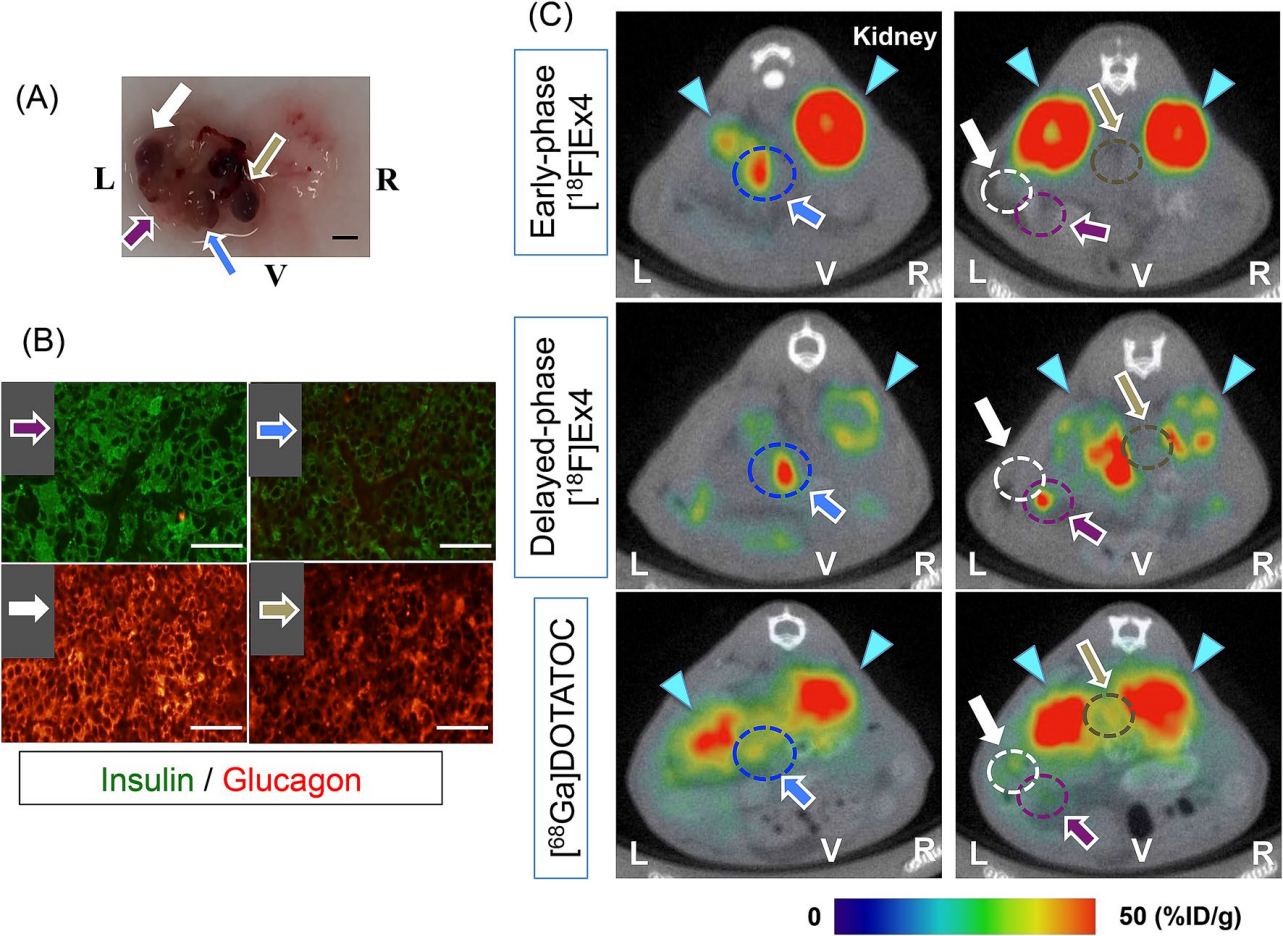
Representative mice images of [^18^F]FB(ePEG12)12-exendin-4 and [^68^Ga]DOTATOC PET/CT. (**A**) Gross image of the pancreas of representative *Pdx1-Cre;Trp53*^*R172H*^*;Rb*^*f/f*^ mouse. A total of four pancreatic tumors were identified. Scale bars indicate 1.0 mm. (**B**) Images of double immunofluorescent staining of insulin and glucagon in each pancreatic tumor. Two tumors showed insulin-positivity, whereas the other two showed insulin negativity and glucagon-positive. Scale bars indicate 100 μm. (**C**) Representative images of [^18^F]FB(ePEG12)12-exendin-4 and [^68^Ga]DOTATOC PET/CT. Arrows and circles with the same color indicate the identical tumor in (**A**–**C**). [^18^F]Ex4; [^18^F]FB(ePEG12)12-exendin-4 PET/CT. Signals from the kidney are represented by light blue arrowheads. %ID/g, uptake values of the region of interest per injected dose of the probe; L, left; R, right; V, ventral.

The *Pdx1-Cre;Trp53*^*R172H*^*;Rb*^*f/f*^ mice with non-fasting blood glucose levels of < 80 mg/dL were enrolled in the comparison study among the [^68^Ga]DOTATOC and the scans at 30- and 120-min post-injection of [^18^F]FB(ePEG12)12-exendin-4 PET/CT. The [^18^F]FB(ePEG12)12-exendin-4 PET/CT was performed approximately a week after a [^68^Ga]DOTATOC PET/CT scan. Three mice completed all the procedures, and no mice died during [^18^F]FB(ePEG12)12-exendin-4 PET/CT scans. The mean mice age was 218.7 ± 13.0 days, and their mean body weight was 30.9 ± 1.6 g. The plasma glucose, insulin and glucagon levels were 46.3 ± 21.3 mg/dL, 2439.7 ± 687.9 pg/mL, and 107.3 ± 19.6 pg/mL, respectively. The plasma insulin per glucose and glucagon per glucose ratios were 67.7 ± 11.1 (/10^7^) and 4.9 ± 2.3 (/10^7^), respectively. The representative mouse images of [^68^Ga]DOTATOC and 30- and 120-min post-injection scans of [^18^F]FB(ePEG12)12-exendin-4 PET/CT are shown in Fig. 4. In this mouse, the two insulin-positive and the other two insulin-negative tumors were identified by histological examination (Fig. 4A,B). Both of the two insulin-positive tumors were identified in 120-min post-injection scan of [^18^F]FB(ePEG12)12-exendin-4 PET/CT, and one of the two was also detected in 30-min post-injection scan of [^18^F]FB(ePEG12)12-exendin-4 and [^68^Ga]DOTATOC PET/CT (Fig. 4C). No insulin-negative tumors were visualized in the [^18^F]FB(ePEG12)12-exendin-4 PET/CT, whereas two insulin-negative tumors were detected in the [^68^Ga]DOTATOC PET/CT.

The results of the comparative PET/CT scans in all the mice are demonstrated in Table 2. A total of nine pancreatic tumor sections were histologically examined; six were insulin-positive tumors and three were insulin-negative tumors, with mean tumor sizes of 1.3 ± 0.3 and 2.0 ± 0.6 mm in diameter, respectively. In 30-min post-injection scan of [^18^F]FB(ePEG12)12-exendin-4 PET/CT, four of the six insulin-positive tumors (66.7%) were detected while no insulin-negative tumors (0%) were visualized. The uptake ratios of the tumor per pancreas were 2.67 ± 0.33 and those of the tumor per kidney were 0.28 ± 0.04. In 120-min post-injection scan of [^18^F]FB(ePEG12)12-exendin-4 PET/CT, five of the six insulin-positive tumors (83.3%) were identified while no insulin-negative tumors (0%) were visualized. The uptake ratios of the tumor per pancreas were 3.67 ± 0.39 (vs. 30-min post-injection, P = 0.14) and those of the tumor per kidney were 0.42 ± 0.06 (vs. 30-min post-injection, P = 0.17). In the early or delayed-phase [^18^F]FB(ePEG12)12-exendin-4 PET/CT, all the insulin-positive tumors (100%) were detected. Conversely, in [^68^Ga]DOTATOC PET/CT, four of the six insulin-positive tumors (66.7%) were detected, whereas all the three insulin-negative tumors (100%) were visualized.

**Table 2:** The comparative results of [^18^F]FB(ePEG12)12-exendin-4 and [^68^Ga]DOTATOC PET/CT.

Tumor	IHC (insulin/glucagon)	Tumor size (mm diameter)	30-min [^18^F]Ex4	120-min [^18^F]Ex4	Combined (30 and 120 min)	[^68^Ga]DOTATOC
Insulin-positive (n = 6)	+ / −	1.3 ± 0.3 mm	66.7% (4/6)	83.3% (5/6)	100% (6/6)	66.7% (4/6)
Insulin-negative (n = 3)	− / +	2.0 ± 0.6 mm	0% (0/3)	0% (0/3)	0% (0/3)	100% (3/3)
*P* value (insulin-positive vs. insulin-negative)		0.25	0.07	0.03*	< 0.01*	0.29
Sensitivity/specificity for detecting insulin-positive tumors (%)		–	66.7/100	83.3/100	100/100	66.7/0

The data are expressed as the mean ± SEM.

*IHC* immunohistochemistry, *[*^*18*^*F]Ex4* [^18^F]FB(ePEG12)12-exendin-4, *PET/CT* Positron emission tomography/Computed tomography, *30-min* PET scans started 30 min after the probe injection, *120-min* PET scans started 120 min after the probe injection; *P < 0.05.

### In vitro internalization of [^18^F]FB(ePEG12)12-exendin-4

**Figure 5:**
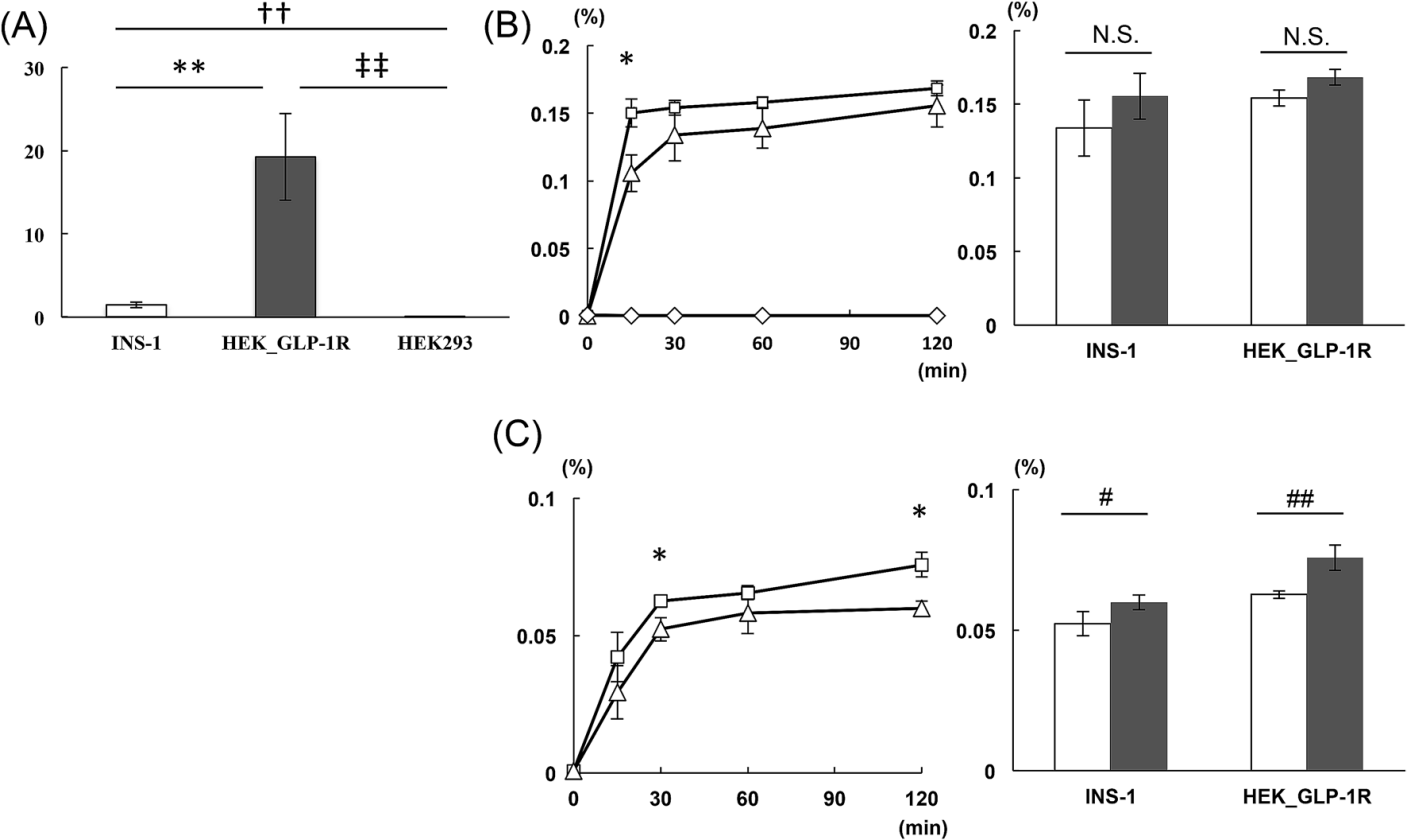
In vitro internalization of [^18^F]FB(ePEG12)12-exendin-4. (**A**) Relative GLP-1R mRNA expression levels in INS-1, HEK293/GLP-1R(+), and HEK293/GLP-1R(−) cells (n = 6 for each group). INS-1, white bar; HEK293/GLP-1R(+), gray bar; HEK293/GLP-1R(−), black bar. **P < 0.01, ^††^P < 0.01, ^‡‡^P < 0.01. (**B**) The changes in whole-cell radioisotope activity per incubated [^18^F]FB(ePEG12)12-exendin-4 probe activities are shown in the left column (n = 6 for each group). The comparisons of whole-cell radioisotope activity per incubated probe activities between 30 and 120 min after incubation with the probe are shown in the right column. (**C**) The changes of cell-internalized radioisotope activity per incubated [^18^F]FB(ePEG12)12-exendin-4 probe activities is shown in the left column (n = 6 for each group). The comparisons of cell-internalized radioisotope activity per incubated probe activities between 30 and 120 min after incubation with the probe are shown in the right column (n = 6 for each group). The data are expressed as the mean ± SEM. INS-1, square; HEK293/GLP-1R(+), triangle; HEK293/GLP-1R(−), rhombi. 30 min, white bar; 120 min, gray bar. ^§^P < 0.05, radioisotope activities per incubated probe activities in INS-1 vs. HEK293/GLP-1R(+) cells. N.S., not significant; ^#^P < 0.05, ^##^P < 0.01.

Further, we investigated time course of probe accumulations in the tumor via whole-cell and cell-internalized radioisotope activities after the indicated varying incubation periods with the labeled ([^18^F]FB(ePEG12)12-exendin-4) probe, using INS-1 and HEK293/GLP-1R(+) cells, in which GLP-1R mRNA expressions were confirmed by quantitative PCR (Fig. 5A). The values of the whole-cell radioisotope activities obtained per incubated probe activities showed rapid increase within the initial 30 min of incubation, followed by a gradual increase up to 120 min in INS-1 and HEK293/GLP-1R(+) cells, whereas HEK293/GLP-1R(−) cells showed no significant probe accumulations during the observation period (Fig. 5B). The values of the cell-internalized radioisotope activities per incubated probe activities increased within 30 min and gradually up to 120 min (Fig. 5C). The cell-internalized radioisotope activities per incubated probe activities after 120 min of incubation were significantly higher than those after 30 min of incubation in both cell lines (INS-1 cells: 0.060 ± 0.003 vs. 0.052 ± 0.004%, p = 0.03; HEK293/GLP-1R(+) cells: 0.076 ± 0.005 vs. 0.063 ± 0.001%, p < 0.01).

## Discussion

This is the preclinical study investigating the utility of [^18^F]FB(ePEG12)12-exendin-4, a novel PEGylated exendin-4-based probe, in PET/CT imaging for detecting insulinoma using both INS-1 xenograft mouse models and *Pdx1-Cre;Trp53*^*R172H*^*;Rb*^*f/f*^ mice, a novel multiple panNEN mouse model, with developed endogenous insulinoma and glucagonoma. In the INS-1 xenograft mouse models, the [^18^F]FB(ePEG12)12-exendin-4 PET/CT clearly visualized INS-1 tumors in the thigh as well as inside the pancreas. In *Pdx1-Cre;Trp53*^*R172H*^*;Rb*^*f/f*^ mice, compared with [^68^Ga]DOTATOC PET/CT, [^18^F]FB(ePEG12)12-exendin-4 PET/CT yielded favorable sensitivity and specificity for insulinoma detection. Besides, [^18^F]FB(ePEG12)12-exendin-4 PET/CT at 120-min post-injection scan demonstrated better pancreatic tumor visualizations than the early-phase scan alone in accordance with the in vitro time-based analysis of probe accumulation.

PET and SPECT imaging techniques with various radiolabeled exendin-4-based probes have been investigated in an attempt to localize insulinoma^14–17,23–25^. Among several radiolabel types, fluorine-18 is one of the most favorable radioisotopes for nuclear medicine imaging owing to its low positron emission energy and potential for high resolution and wide use in today’s clinical settings^16,18^. However, most previous reports on ^18^F-labeled exendin analogs demonstrated relatively high non-specific uptakes in preclinical studies, mostly due to the labeling methodologies^16,26,27^. Notably, PEGylation became an alternative owing to its ability to increase the molecular weight of the probe and its stability in circulation as well as improvement of the probe uptake by the tumor cells and leakage prevention from the tumor^20,21,28^. Therefore, we considered examining [^18^F]FB(ePEG12)12-exendin-4 as a detection probe for insulinoma. [^18^F]FB(ePEG12)12-exendin-4 demonstrated reasonable stability in vivo and sustained pancreatic uptakes with rapid kidney clearance in biodistribution study using wild type mice (Table 1). In comparison with previously reported non-PEGylated probes, [^18^F]FB-exendin-4 and ^18^F-[Nle^14^, Lys^40^]exendin-4, [^18^F]FB(ePEG12)12-exendin-4 showed clearly higher uptakes on pancreas^28,29^. Although ^18^F-[Nle^14^, Lys^40^]exendin-4 reduced kidney uptakes compared with [^18^F]FB-exendin-4 and other radiometal-labeled exendin derivatives, [^18^F]FB(ePEG12)12-exendin-4 demonstrated comparably low kidney uptakes and rapid clearance^29^. In addition, [^18^F]FB(ePEG12)12-exendin-4 PET/CT was demonstrated to be a useful imaging approach because it successfully visualized INS-1 xenografts with high uptake ratios of the tumor compared to pancreas (Fig. 1). In comparison with [^18^F]FB-exendin-4, our PEGylated probe showed improved tumor uptake and higher contrast with surrounding organs including kidneys, although the available data obtained from the previous report regarding [^18^F]FB-exendin-4 is limited^28^. Our probe yielded higher uptake values on tumor than on kidneys, whereas the uptake ratios of tumor per kidney of [^18^F]FB-exendin-4 were reported as less than 1.0^28^. Such differences of probe kinetics might lead to the improvement of tumor visualization on PET imaging by introducing the PEG linker. Further experiments wherein head-to-head comparison between the analogues with and without PEG linker would produce conclusive information on the role of the PEG linker and its influence on the organ distribution.

In addition to PEGylation, various modifications on ^18^F-labeled exendin-4 have been implemented to reduce nonspecific uptakes including kidney with improvement of tumor uptakes^18,19,27,30–32^. The tumor uptake values of our PEGylated probe at 60-min post-injection in the resected organs of mice bearing INS-1 xenografts (Right thigh: 37.3 ± 4.5%ID/g, Inside pancreas: 37.7 ± 4.4%ID/g) showed comparable or higher values than those of other ^18^F-labeled non-PEGylated modified exendin-4 probes; 17.9 ± 1.4%ID/g in biodistribution study of [^18^F]aluminum fluoride (AlF)-1,4,7‑triazacyclononanetriacetic acid (NOTA)-maleimide (MAL)-Cys^40^-exendin-4^30^, 33.2 ± 4.8%ID/g in biodistribution study of [^18^F]fluoropentylmaleimide (FPenM)-[Cys^40^]exendin-4^31^, 25.3 ± 3.4%ID/g in imaging study of [^18^F]fluorobenzamide (FBEM)-[Cys^40^]exendin-4^27^, and 19.6 ± 4.6%ID/g in imaging study of [^18^F]fluoronicotinamide (FNEM)-[Cys^40^]exendin-4^32^. As for the uptake ratios of tumor per kidney, our PEGylated probe at 60-min post-injection in the resected organs of mice bearing INS-1 xenografts reached 3.30 ± 0.36 (right thigh) and 3.44 ± 1.00 (inside pancreas). Although the uptake ratios of tumor per kidney in most of the ^18^F-labeled exendin-4 probes were reported as less than 1.0^18^, some modifications such as [^18^F]FPenM-[Cys^40^]exendin-4 and [^18^F]FNEM-[Cys^40^]exendin-4 successfully demonstrated improved uptake ratios of tumor per kidney that were comparable to our PEGylated probe^31,32^. In view of insulinoma visualization, the advantages and disadvantages of [^18^F]FB(ePEG12)12-exendin-4 were summarized in Table 3.

**Table 3:** The advantages and disadvantages of [^18^F]FB(ePEG12)12-exendin-4 in comparison with other representative non-PEGylated probes.

Our probe [^18^F]FB(ePEG12)12-exendin-4	Advantages	Disadvantages
Vs [^18^F]FB-exendin-4^28^ ^18^F-[Nle^14^, Lys^40^]exendin-4^29^	Higher tumor uptake^a^ Higher tumor/kidney uptake^a^ Lower kidney uptake and rapid kidney clearance Higher pancreas uptake/lower uptakes on other abdominal organs	–
Vs [^18^F]FBEM-[Cys^40^]exendin-4^27^ [^18^F]AlF-1,4,7‑NOTA-MAL-Cys^40^-exendin-4^30^ [^18^F]FPenM-[Cys^40^]exendin-4^31^ [^18^F]FNEM-[Cys^40^]exendin-4^32^	Higher or comparable tumor uptake Comparable tumor/kidney uptake High uptake on tumor implanted inside pancreas or tumor spontaneously developed in pancreas/high contrast with intact pancreas Lower or comparable liver uptake retention	Relatively long synthesis time (120 min)

^a^The comparison was performed with [^18^F]FB-exendin-4. The data regarding tumor uptakes of ^18^F-[Nle^14^, Lys^40^]exendin-4 is not available.

Identifying a suitable mouse model for the preclinical evaluations of imaging probes in the localization of insulinoma remains an issue. INS-1 xenograft mouse models have been conventionally used for this purpose. Indeed, in most preclinical studies, using radiolabeled exendin-4-based probes for subcutaneous and/or intramuscular transplantation of INS-1 cells have been accomplished^18^. However, the visualization of INS-1 xenografts and tumor probe uptake were affected by their transplantation site and circumstances, which might be largely different in the case of endogenous insulinomas. As a mouse model of endogenous insulinoma, a mouse strain with rat insulin gene-2 promoter used to drive transgenic expression of simian virus 40 large T antigen (Rip-Tag2) was applied in a limited number of the previous studies^11,33^. Although Rip-Tag2 mice are well-studied endogenous insulinoma model, their tumorigenesis is limited to pancreatic β cells^34^. We have recently established *Pdx1-Cre;Trp53*^*R172H*^*;Rb*^*f/f*^ mice, which develop endogenous well-differentiated insulinoma as well as glucagonoma with full penetrance^22^. We thought that this mouse strain could be the useful model for differential imaging evaluation of our novel probe in functional panNENs. In the immunohistochemical study, insulin-positive pancreatic tumors exhibited positivity on [^18^F]FB(ePEG12)12-exendin-4 PET/CT imaging and showed significant uptake (Fig. 4, Table 2); however, immunohistochemically, insulin-negative and glucagon-positive pancreatic tumors were negative on [^18^F]FB(ePEG12)12-exendin-4 PET/CT images but positive on [^68^Ga]DOTATOC PET/CT images, suggesting the selectivity of our probe. These results are consistent with those of GLP-1R and SSTR subtype 2 (SSTR2) mRNA expressions, in which insulin-positive tumors showed significantly high GLP-1R and relatively low SSTR2 mRNA expression levels (Fig. 2). Therefore, *Pdx1-Cre;TRp53*^*R172H*^*;Rb*^*f/f*^ is a suitable model in preclinical investigations of exendin-4-based probes for the specific detection of insulinoma.

In our investigation of [^18^F]FB(ePEG12)12-exendin-4 PET/CT scans of *Pdx1-Cre;Trp53*^*R172H*^*;Rb*^*f/f*^ mice, the scans at 30- and 120-min post-injection demonstrated different visualization states and probe uptake, which led to the best detection ratios in combined analyses of both scans (Fig. 4, Table 2). The scan at 120-min post-injection showed higher detection ratios among insulin-positive tumors than at 120-min post-injection. As previously reported, ^18^F-labeled exendin-4-based probes have features of more rapid clearance from the kidney than other radiometal labeled probes^18,35,36^. In addition, our in vitro analyses showed the internalization and stable retention of [^18^F]FB(ePEG12)12-exendin-4 probe in the cells; whole-cell and cell-internalized probe accumulated even at 120 min of incubation following the rapid increase within 30 min (Fig. 5B,C). These characteristics might contribute to a better contrast between the tumors and the surrounding organs in delayed-phase scans. Michalski et al. reported that a single 60-min early-phase [Nle14,Lys40(Ahx-DOTA-Ga-68)NH2]exendin-4 ([^68^Ga]DOTA-exendin-4) PET/CT scan is sufficient for localizing insulinoma, and dual-time-point scans at 60 and 120 min after injection of [^68^Ga]DOTA-exendin-4 had no additional diagnostic value^39^. However, their scan at 60-min post-injection might not be an early-phase one. Moreover, a longer half-life than gallium-68 (109.8 min vs. 67.7 min) and different chemical modifications could affect the optimized time of scans. Although our results suggested that the scan at 120-min post-injection and/or repeated scans might have potential benefit on insulinoma detecting in *Pdx1-Cre;Trp53*^*R172H*^*;Rb*^*f/f*^ mice, further clinical investigations are warranted to confirm the optimal scan protocol for detecting insulinoma using our probe.

Finally, our study has limitations; the relatively small number of mice used in the imaging analysis might impede more definitive conclusions about the protocol being optimal for our analysis of the [^18^F]FB(ePEG12)12-exendin-4 PET/CT scans for the detection of insulinoma. However, since most of the *Pdx1-Cre;Trp53*^*R172H*^*;Rb*^*f/f*^ mice develop insulinoma only at the age of over 180 days and those mice die within 2 weeks after presenting with hypoglycemia as previously reported^22^, it is challenging to employ a larger number of the mice to complete our comparative protocol between [^18^F]FB(ePEG12)12-exendin-4 and [^68^Ga]DOTATOC PET/CT scans. In addition, this is a preclinical study and further clinical investigations are needed in order to confirm the utility and the optimal scan protocol of [^18^F]FB(ePEG12)12-exendin-4 PET/CT. Moreover, a dynamic study for 120–180 min might provide additional information on the whole body pharmacokinetics and reveal the tumor uptake kinetics and kidney clearance that would allow optimization of the scanning time point in terms of the image contrast. Further investigations including comparative studies with other exendin-4 based probes are warranted to confirm any benefits over other chemical modifications of exendin-4.

In conclusion, employing [^18^F]FB(ePEG12)12-exendin-4 PET/CT for the detection of insulinoma yielded favorable sensitivity and specificity in the INS-1 xenograft mouse models and the *Pdx1-Cre;Trp53*^*R172H*^*;Rb*^*f/f*^ mice with endogenous insulinoma and glucagonoma.

## Materials and methods

### Animals

Male ddY and BALB/c slc-*nu*/*nu* mice were purchased from SLC Japan (Hamamatsu, Japan). Male *Pdx1-Cre;TRp53*^*R172H*^*;Rb*^*f/f*^ mice were obtained from the established colonies^22^. The mice enabled us to examine the PET probe’s specificity to both insulin-positive and -negative neuroendocrine neoplasms in the intact pancreas at one scan. We measured non-fasting blood glucose levels of *Pdx1-Cre;Trp53*^*R172H*^*;Rb*^*f/f*^ mice weekly using the glucose oxidase method (GT-1670; Arkray, Kyoto, Japan) and only the mice with blood glucose levels of < 80 mg/dL were enrolled in this study because such mice were expected to develop insulinoma^22^. All the mice were housed in a temperature-maintained environment under conditions of 14:10 light–dark cycles with free access to water and food unless otherwise stated.

This animal study was approved by the animal care and use committee, Kyoto University Graduate School of Medicine (Med kyo 20218, 19246, 18246). All methods were performed in accordance with the relevant guidelines and regulations including the ARRIVE guidelines.

### Measurement of mouse plasma glucose, insulin, and glucagon levels

The plasma glucose, insulin, and glucagon levels were measured using the Glucose CII Test Wako (Wako Pure Chemical Industries, Ltd., Osaka, Japan), an insulin ELISA kit (Ultra-Sensitive Mouse Insulin ELISA Kit; Morinaga Institute of Biological Science, Japan), and a glucagon ELISA kit (Cat# 10-1281-01; Mercodia, Sweden), respectively.

### Cell preparations and cultures

Rat insulinoma INS-1 cells were cultured in RPMI 1640 medium as previously described^37^. Human embryonic kidney (HEK) 293 cells [HEK293/GLP-1R(−)] were obtained from ATCC and cultured in Dulbecco’s modified Eagle’s medium containing 11.1 mM glucose (D5796; Sigma, CA, USA) supplemented with 10% fetal bovine serum (FBS), 1 mM sodium pyruvate, 0.060 mM 2-mercaptoethanol, 100 units/mL penicillin, and 100 μg/mL streptomycin in a humidified atmosphere containing 5% CO_2_ at 37 °C. HEK293 cells expressing human GLP-1R [HEK293/GLP-1R(+)] were obtained through stable transfection with CAGIP encoding human GLP-1R (exon1–13) cloned into EcoRV site of pBSIIKS(−) (Stratagene, La Jolla, CA, USA).

### Ribonucleic acid isolation and real-time polymerase chain reaction

Total ribonucleic acid (RNA) was extracted from each of three samples, cultured cells, snap-frozen pancreas, and enucleated pancreatic tumors of *Pdx1-Cre;Trp53*^*R172H*^*;Rb*^*f/f*^ mice (n = 10) using an RNA isolation kit (Qiagen, Valencia, CA, USA)^38^, following the manufacturer’s instructions. For complementary deoxyribonucleic acid synthesis, 1 μg total RNA from each sample was reverse transcribed using a High Capacity RNA-to-cDNA Kit (Applied Biosystems, Alameda, CA, USA), following the manufacturer’s protocol. SYBR Green polymerase chain reaction (PCR) Master Mix (Applied Biosystems) was applied for quantitative real-time PCR using an ABI StepOne-Plus Real-Time PCR System (Applied Biosystems, CA, USA). The signals of the products were standardized against glyceraldehyde 3-phosphate dehydrogenase (GAPDH) signals of each sample. Primer pairs for PCR are as follows: mice GAPDH, 5′-AAATGGTGAAGGTCGGTGTG-3′ and 5′-TCGTTGATGGCAACAATCTC-3′; mice GLP-1R, 5′-CAACCGGACCTTTGATGACTA-3′ and 5′-GCTGTGCAGAACCGGTACAC-3′; rat GAPDH, 5′-TGATTCTACCCACGGCAAGTT-3′ and 5′-TGATGGGTTTCCCATTGATGA-3′; human GAPDH, 5′-GGATTTGGTCGTATTGGG-3′ and 5′-GGAAGATGGTGATGGGATT-3′; human GLP-1R, 5′-TTCTGCAACCGGACCTTCGA-3′ and 5′-ATGAGTGTCAGCGTGGACTTG-3′.

### Synthesis of [^18^F]FB(ePEG12)12-exendin-4

**Figure 6:**
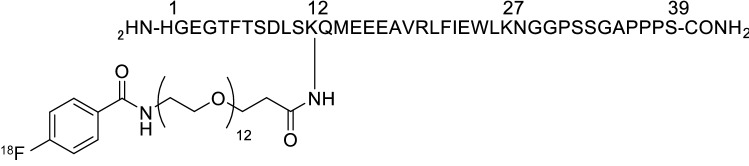
Chemical structure of [^18^F]FB(ePEG12)12-exendin-4. A chemical structure of [^18^F]FB(ePEG12)12-exendin-4 is shown. Exendin-4 was conjugated with polyethylene glycol (PEG) and [^18^F]succinyl-4-fluorobenzoate (SFB).

*N*-Succinimidyl-4-[^18^F]fluorobenzoate ([^18^F]SFB) was prepared, as previously described^12^. A solution of peptide precursors, pre(ePEG12)12-exendin-4 (KNC Laboratories Co. Ltd., Kobe, Japan) in acetonitrile/buffer containing 0.5 M dipotassium hydrogen phosphate (Fujifilm Wako Pure Chemical Corporation, Osaka, Japan) was added to the reaction vessel of [^18^F]SFB. The reaction mixture was incubated at 60 °C for 15 min. Next, 4-metylpiperidine (Fujifilm Wako Pure Chemical Corporation, Osaka, Japan) was added, and the solution was incubated further at 60 °C for 10 min. The synthesized [^18^F]FB(ePEG12)12-exendin-4 (Fig. 6) was purified by reversed-phase high-performance liquid chromatography (RP-HPLC) using a YMC Triart C8 column (10 × 250 mm, 5 μm) (YMC, Kyoto, Japan) and loaded onto an activated Sep-Pak C18 (Sep-Pak light C18 cartridge: Waters Co., Milford, MA, USA). Finally, [^18^F]FB(ePEG12)12-exendin-4 was eluted with ethanol (Fujifilm Wako Pure Chemical Corporation, Osaka, Japan).

### Ex-vivo biodistribution study of [^18^F]FB(ePEG12)12-exendin-4

Male 6-week-old ddY mice were used in biodistribution study. [^18^F]FB(ePEG12)12-exendin-4 (0.74 MBq/mouse) was administered via the tail vein. The mice were sacrificed by cervical dislocation at 5, 15, 30, 60, 150, and 360 min after administration (n = 4 for each time point), followed by immediate resection of each organ including the pancreas, kidney, liver, spleen, lung, and blood. The radioactivity measurements of the resected organs were performed using an auto-well γ-counter (COBRA II, Perkin Elmer, Waltham, MA, USA).

### Generation of the INS-1 xenograft mice model

For each eight-week-old male BALB/c slc-*nu*/*nu* mice (n = 5), 2.0 × 10^5^–1.0 × 10^7^ INS-1 cells in 100 μL of phosphate-buffered saline (PBS) were subcutaneously inoculated into the right thigh, as previously described^37^. Similarly, 5.0 × 10^5^ INS-1 cells were inoculated into the pancreas of each eight-week-old male BALB/c slc-*nu*/*nu* mice (n = 3).

### [^18^F]FB(ePEG12)12-exendin-4 PET/CT study

[^18^F]FB(ePEG12)12-exendin-4 PET/CT scans were performed in INS-1 tumor-bearing mice at 4 weeks after injection. [^18^F]FB(ePEG12)12-exendin-4 (0.148 MBq/mouse) was injected via the tail vein, and PET/CT imaging was performed after 30 min postinjection under 2.0% isoflurane anesthesia using a Triumph LabPET12/SPECT4/CT (TriFoil Imaging Inc., Chatsworth, CA, USA), as previously described^12^. The image analysis including maximum intensity projection (MIP) reconstructions was performed using the Amira software version 5.6.0 (FEI Visualization Sciences Group, Düsseldorf, Germany). Subsequently, the mice were sacrificed by cervical dislocation 60 min after injection, followed by immediate resection of the INS-1 tumors, and radioactivity measurements of the resected tumors were performed using an auto-well γ-counter (COBRA II, Perkin Elmer, Waltham, MA, USA). The radioactivity measurements were decay-corrected to the time of injection. Then, the tissue and tumor radioactivity levels were expressed as their uptake values per injected dose of the probe (%ID/g)^12,23,39^.

### PET/CT study in *Pdx1-Cre;Trp53*^*R172H*^*;Rb*^*f/f*^ mice

The *Pdx1-Cre;Trp53*^*R172H*^*;Rb*^*f/f*^ mice with non-fasting blood glucose levels of < 80 mg/dL were used for ^68^Ga-labeled 1,4,7,10-tetraazacyclododecane-N,N′,N″,N″-tetraacetic acid-d-Phe^1^-Tyr^3^-octreotide ([^68^Ga]DOTATOC) PET/CT; [^68^Ga]DOTATOC was synthesized according to the previous report ^40^. PET scans were performed after 30 min of injection of the labeled substance via tail vein (10 MBq/mouse) under 2.0% isoflurane anesthesia using a Triumph LabPET12/SPECT4/CT (TriFoil Imaging Inc., Chatsworth, CA, USA)^41^. After a week of [^68^Ga]DOTATOC PET/CT scans, [^18^F]FB(ePEG12)12-exendin-4 PET/CT imaging was performed in two phases, 30 min (early-phase) and 120 min (delayed-phase) postinjection, of the [^18^F]FB(ePEG12)12-exendin-4 tail vein (0.148 MBq/mouse) under 2.0% isoflurane anesthesia using a Triumph LabPET12/SPECT4/CT. The image analysis of [^68^Ga]DOTATOC and [^18^F]FB(ePEG12)12-exendin-4 PET/CT were performed after all the PET/CT scans were completed (n = 3). The data from only the mice completing the protocol were analyzed since the most of the *Pdx1-Cre;Trp53*^*R172H*^*;Rb*^*f/f*^ mice developing insulinoma die within 2 weeks after presenting with hypoglycemia as previously reported^22^.

### Histological analysis of *Pdx1-Cre;Trp53*^*R172H*^*;Rb*^*f/f*^ mice

After completion of the PET/CT procedures, mice were sacrificed by cervical dislocation, followed by immediate resection of the pancreas. Tissues were spread on filter paper and immediately fixed in 10% formalin at 4 °C. For the analysis of pancreatic tumors, serial formalin-fixed paraffin-embedded sections (4-μm thickness) were stained with anti-insulin and anti-glucagon antibody, as previously reported^39^. Briefly, primary rabbit polyclonal antibody was used for insulin detection at an antibody dilution of 1:100 (Cat# sc-9168; Santa Cruz Biotechnology, USA) and a mouse monoclonal antibody glucagon detection at an antibody dilution of 1:100 (Cat# ab10988; Abcam, USA). The secondary antibodies utilized for the assay were Alexa Fluor 488 goat anti-rabbit antibody at 1:200 dilution (Cat# A-11008; Thermo Fisher Scientific, USA) and Alexa Fluor 546 goat anti-mouse antibody at 1:200 dilution (Cat# A-11030; Thermo Fisher Scientific, USA), respectively. The slides were prepared for analyses under a fluorescence microscope (BZ-X700; Keyence, Osaka, Japan).

### In vitro internalization study of [^18^F]FB(ePEG12)12-exendin-4

Time-based internalization of [^18^F]FB(ePEG12)12-exendin-4 was investigated using INS-1, HEK293/GLP-1R(−), and HEK293/GLP-1R(+) cells (n = 6 for each group), as previously described^42^. The cells were incubated with 1.48 MBq of [^18^F]FB(ePEG12)12-exendin-4 for different periods (0, 15, 30, 60, and 120 min). Subsequently, the cells were rinsed twice with chilled PBS; then, whole-cell and cell-internalized radioisotope activities were measured by the COBRA II γ-counter. The radioactivity measurements were decay-corrected to the time of incubation. For the measurement of the cell-internalized activity, the surface-bound radioactivity was removed by incubation with acid stripping buffer (50 mmol/L glycine–HCl/100 mmol/L NaCl, pH 2.8) at room temperature for 20 min. Then, the internalized activity was measured relative to the total radioactivity added.

### Statistical analysis

All data were expressed as the mean ± SEM. Statistical analyses were performed using a one-way analysis of variance with the Tukey–Kramer post hoc test and Student’s or Welch’s t-test. P-values of < 0.05 were considered statistically significant. The statistical analysis was carried out using SPSS Statistics 24 software (IBM Corp., Armonk, NY, USA) and JMP Pro^®^, version 15.1 (SAS Institute Inc., Cary, NC, USA).

## References

[1] Serveice FJ, McMahon MM, O’Brien PC, Ballard DJ (1991). Functioning insulinoma-incidence, recurrence, and long-term survival of patients: A 60-year study. Mayo Clin. Proc..

[2] Grant CS (2005). Insulinoma. Best Pract. Res. Clin. Gastroenterol..

[3] Murakami T, Yabe D, Inagaki N (2018). Case 23–2018: A man with episodes of confusion and hypoglycemia. N. Engl. J. Med..

[4] Imamura M (2010). Recent standardization of treatment strategy for pancreatic neuroendocrine tumors. World J. Gastroenterol..

[5] Placzkowski KA (2009). Secular trends in the presentation and management of functioning insulinoma at the Mayo Clinic, 1987–2007. J. Clin. Endocrinol. Metab..

[6] Mehrabi A (2014). A systematic review of localization, surgical treatment options, and outcome of insulinoma. Pancreas.

[7] Hatoko T (2020). Low-dose selective arterial calcium stimulation test for localizing insulinoma: A single-center experience on five consecutive cases. Intern. Med..

[8] Reubi JC, Waser B (2003). Concomitant expression of several peptide receptors in neuroendocrine tumours: Molecular basis for in vivo multireceptor tumour targeting. Eur. J. Nucl. Med. Mol. Imaging..

[9] Bertherat J (2003). Somatostatin receptors 2 and 5 are the major somatostatin receptors in insulinomas: An in vivo and in vitro study. J. Clin. Endocrinol. Metab..

[10] Reubi JC, Körner M, Waser B, Mazzucchelli L, Guillou L (2004). High expression of peptide receptors as a novel target in gastrointestinal stromal tumours. Eur. J. Nucl. Med. Mol. Imaging..

[11] Wild D (2006). [Lys40(Ahx-DTPA-111In)NH2]exendin-4, a very promising ligand for glucagon-like peptide-1 (GLP-1) receptor targeting. J. Nucl. Med..

[12] Kimura H (2018). Evaluation of 18 F-labeled exendin(9–39) derivatives targeting glucagon-like peptide-1 receptor for pancreatic β-Cell Imaging. Bioorg. Med. Chem..

[13] Murakami T, Fujimoto H, Inagaki N (2021). Non-invasive beta-cell imaging: Visualization, quantification, and beyond. Front. Endocrinol. (Lausanne)..

[14] Christ E (2009). Glucagon-like peptide-1 receptor imaging for localization of insulinomas. J. Clin. Endocrinol. Metab..

[15] Christ E (2013). Glucagon-like peptide-1 receptor imaging for the localisation of insulinomas: A prospective multicentre imaging study. Lancet Diabetes Endocrinol..

[16] Antwi K (2018). Comparison of glucagon-like peptide-1 receptor (GLP-1R) PET/CT, SPECT/CT and 3T MRI for the localisation of occult insulinomas: Evaluation of diagnostic accuracy in a prospective crossover imaging study. Eur. J. Nucl. Med. Mol. Imaging..

[17] Luo Y (2016). Glucagon-like peptide-1 receptor PET/CT with 68Ga-NOTA-exendin-4 for detecting localized insulinoma: A prospective cohort study. J. Nucl. Med..

[18] Jansen TJP (2019). Exendin-4 analogs in insulinoma theranostics. J. Labelled Comp. Radiopharm..

[19] Refardt J (2020). Theranostics in neuroendocrine tumors: An overview of current approaches and future challenges. Rev. Endocr. Metab. Disord..

[20] Harris JM, Chess RB (2003). Effect of pegylation on pharmaceuticals. Rev. Drug Discov..

[21] Tornesello AL, Buonaguro L, Tornesello ML, Buonaguro FM (2017). New insights in the design of bioactive peptides and chelating agents for imaging and therapy in oncology. Molecules.

[22] Yamauchi Y (2020). Rb and p53 execute distinct roles in the development of pancreatic neuroendocrine tumors. Cancer Res..

[23] Kimura H (2017). Development of 111 In-labeled exendin(9–39) derivatives for single-photon emission computed tomography imaging of insulinoma. Bioorg. Med. Chem..

[24] Sowa-Staszczak A (2016). 99mTc labeled glucagon-like peptide-1-analogue (99mTc-GLP1) scintigraphy in the management of patients with occult insulinoma. PLoS One..

[25] Eriksson O (2014). Detection of metastatic insulinoma by positron emission tomography with [(68)ga]exendin-4-a case report. J. Clin. Endocrinol. Metab..

[26] Velikyan I, Eriksson O (2020). Advances in GLP-1 receptor targeting radiolabeled agent development and prospective of theranostics. Theranostics..

[27] Kiesewetter DO (2012). 18F-radiolabeled analogs of exendin-4 for PET imaging of GLP-1 in insulinoma. Eur. J. Nucl. Med. Mol. Imaging..

[28] Wu H (2013). 18F-radiolabeled GLP-1 analog exendin-4 for PET/CT imaging of insulinoma in small animals. Nucl. Med. Commun..

[29] Mikkola K (2016). Low kidney uptake of GLP-1R-targeting, beta cell-specific PET tracer, 18 F-labeled [Nle 14, Lys 40]exendin-4 analog, shows promise for clinical imaging. EJNMMI Res..

[30] Kiesewetter DO (2012). Evaluation of an [(18)F]AlF-NOTA analog of Exendin-4 for imaging of GLP-1 receptor in insulinoma. Theranostics..

[31] Yue X (2013). Development of a new thiol site-specific prosthetic group and its conjugation with [Cys(40)]-exendin-4 for in vivo targeting of insulinomas. Bioconjug. Chem..

[32] Yue X (2014). One-pot two-step radiosynthesis of a new (18)F-labeled thiol reactive prosthetic group and its conjugate for insulinoma imaging. Mol. Pharm..

[33] Mukai H, Wada Y, Watanabe Y (2013). The synthesis of 64Cu-chelated porphyrin photosensitizers and their tumor-targeting peptide conjugates for the evaluation of target cell uptake and PET image-based pharmacokinetics of targeted photodynamic therapy agents. Ann. Nucl. Med..

[34] Wild D (2010). Exendin-4-based radiopharmaceuticals for glucagonlike peptide-1 receptor PET/CT and SPECT/CT. J. Nucl. Med..

[35] Hanahan D (1985). Heritable formation of pancreatic beta-cell tumours in transgenic mice expressing recombinant insulin/simian virus 40 oncogenes. Nature.

[36] Xu Y (2014). Insulinoma imaging with glucagon-like peptide-1 receptor targeting probe (18)F-FBEM-Cys (39)-exendin-4. J. Cancer Res. Clin. Oncol..

[37] Michalski K (2020). Detection of insulinomas using dual-time-point 68Ga-DOTA-Exendin 4 PET/CT. Clin. Nucl. Med..

[38] Murakami T (2020). Association of glucagon-like peptide-1 receptor-targeted imaging probe within vivo glucagon-like peptide-1 receptor agonist glucose-lowering effects. J. Diabetes Investig..

[39] Murakami T (2019). Noninvasive evaluation of GPR119 agonist effects on β-cell mass in diabetic male mice using ^111^In-exendin-4 SPECT/CT. Endocrinology.

[40] Nakamoto Y (2016). Clinical efficacy of dual-phase scanning using (68)Ga-DOTATOC-PET/CT in the detection of neuroendocrine tumours. Clin. Radiol..

[41] Stelter L (2008). An orthotopic model of pancreatic somatostatin receptor (SSTR)-positive tumors allows bimodal imaging studies using 3T MRI and animal PET-based molecular imaging of SSTR expression. Neuroendocrinology.

[42] He Y (2009). The antiproliferative effects of somatostatin receptor subtype 2 in breast cancer cells. Acta Pharmacol. Sin..

